# Risk Stratification in Predicting Cardiogenic Shock 30-Day Mortality admitted to ICU: beyond the Prognostic Performance of SHARC and SCAI Classifications

**DOI:** 10.1016/j.aicoj.2026.100090

**Published:** 2026-05-20

**Authors:** Pierre-Grégoire Guinot, Thomas Hanquiez, Maxime Nguyen, Osama Abou-Ara, Laurent Leborgne, Yazine Mahjoub, Bélaïd Bouhemad, Christophe Beyls, For the PROMIX study group

**Affiliations:** a Department of Anesthesiology and Intensive Care, Dijon University Hospital, F-21000 Dijon, France; b University of Burgundy Europe, LNC UMR1231, F-21000 Dijon, France; c INSERM, LNC UMR1231, F-21000 Dijon, France; d FCS Bourgogne-Franche Comté, LipSTIC LabEx, F-21000 Dijon, France; e Department of Cardiology, Amiens University Hospital, 80000 Amiens, France; f Department of Anesthesiology and Intensive Care, Amiens University Hospital, 80000 Amiens, France; g UR UPJV 7518 SSPC (Simplification of Care of Complex Surgical Patients) Research Unit, University of Picardie Jules Verne, 80000 Amiens, France

**Keywords:** Cardiogenic shock, SHARC classification, Mortality, Risk stratification, Cardiac intensive care unit, Post cardiotomy

## Abstract

**Background:**

Cardiogenic shock (CS) remains a leading cause of mortality in intensive care units (ICU) despite advances in management. The Shock Academic Research Consortium (SHARC) classification provides an etiological framework complementing severity-based systems such as the Society for Cardiovascular Angiography and Interventions (SCAI) classification. This study evaluated the association between SHARC classes and 30-day mortality in a contemporary ICU population and compared their prognostic value with SCAI classification.

**Methods:**

This bicentric retrospective cohort study included 1366 consecutive adults admitted with CS. Patients were classified according to SHARC criteria: acute myocardial infarction (AMI)-CS (n = 303, 22.5%), heart failure (HF)-CS (n = 56, 5.0%), secondary-CS (n = 433, 29.9%), and post-cardiotomy-CS (n = 574, 42.7%). The primary outcome was 30-day all-cause mortality. Multivariable logistic regression models assessed independent predictors of mortality and comparative prognostic performance using Akaike Information Criterion, Bayesian Information Criterion, and discrimination indices.

**Results:**

Thirty-day mortality varied significantly across SHARC classes: AMI-CS (48.7%), HF-CS (45.6%), secondary-CS (39.0%), and post cardiotomy-CS (17.3%) (*p* < 0.001). Multivariable analysis identified age, epinephrine use, vasopressin use, arterial lactate, right ventricular dysfunction, and SHARC classification as independent mortality predictors (all P < 0.05). Adding SHARC classification to clinical variables provided greater incremental prognostic value (ΔAIC = −48.3; AUC=0.734; R^2^ = 0.198) than SCAI classification alone (ΔAIC = −20.0; AUC = 0.723; R^2^ = 0.172). The combined model integrating SHARC, SCAI, and hemodynamic parameters demonstrated the best performance (AUC = 0.819; R^2^ = 0.355).

**Conclusions:**

SHARC etiological classification provides complementary discrimination for 30-day mortality compared with SCAI staging alone, suggesting that shock pathophysiology influences patient outcomes. Integration of both classifications may improve risk stratification and enable more personalized therapeutic approaches in cardiogenic shock management.

## Graphical abstract

**Figure:**
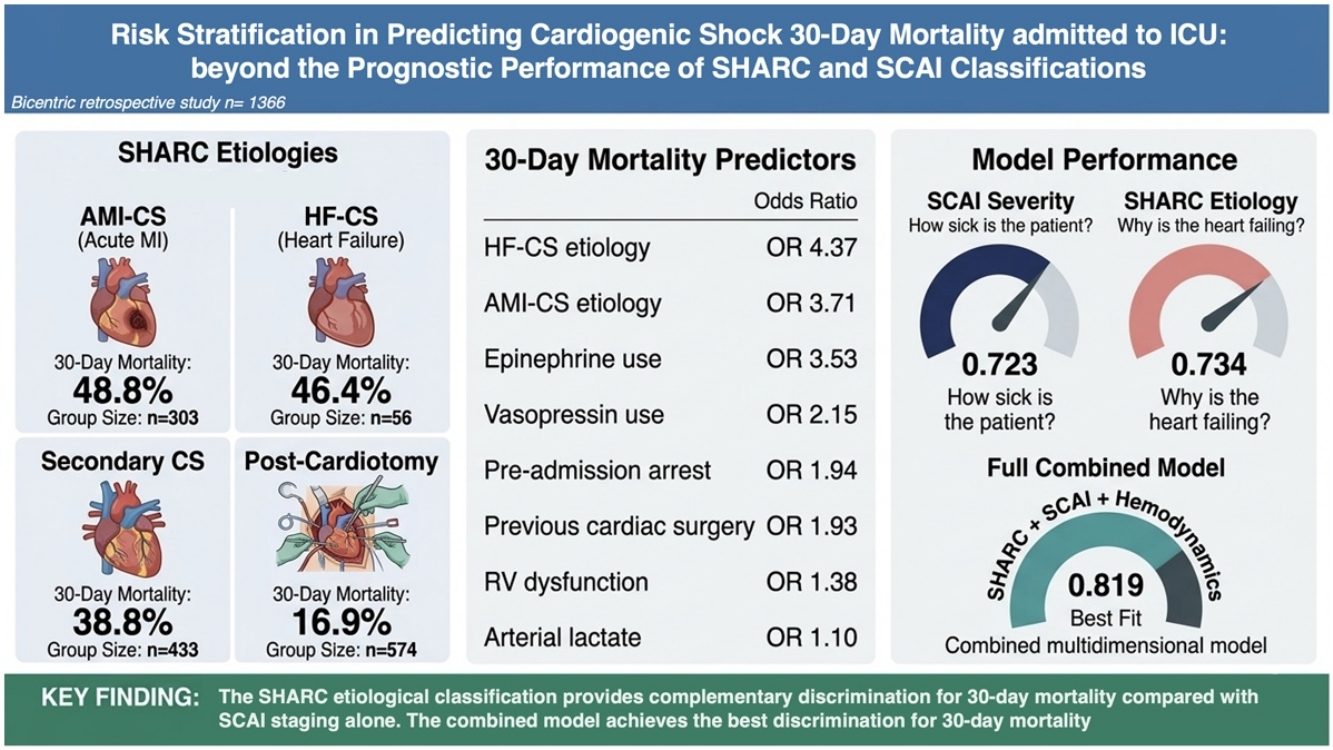

**Keys perspective**

This study demonstrates that combining the SHARC etiological classification, the SCAI severity staging, and the intensity of hemodynamic therapeutic support provides a comprehensive and clinically meaningful characterization of cardiogenic shock. The SCAI stage remains essential for *rapid bedside assessment of severity* and *initial triage*, while the SHARC classification clarifies *why* the patient is in shock, and the type and intensity of hemodynamic support reflect *how severe the shock is*. Integrating these dimensions allows clinicians to quickly identify both the underlying cause and the degree of circulatory failure at ICU admission. This multidimensional approach should support early and appropriate escalation strategies, optimizes resource allocation including timely use of mechanical circulatory support, and enhances interdisciplinary communication between cardiac and intensive care teams. Beyond prognostication, it provides a structured framework for tailoring therapeutic intensity and harmonizing patient selection in future shock trials.

## Introduction

Cardiogenic shock (CS) remains a major concern in intensive care units with high mortality rates ranging between 30 to 50% depending on etiology and high morbidity [1,2]. Despite advancements in etiological treatment and critical care management mortality remains critical, highlighting the need for improved risk stratification and phenotype-specific therapeutic strategies [3]. The heterogeneity of CS has long been recognized as a barrier to both clinical management and research progress [4,5]. Two complementary classification systems have been described to grade and treat in response the patients according to the main etiology and to the severity of shock. The Society for Cardiovascular Angiography and Interventions (SCAI) classification provides a severity-based staging system (A to E) that has been widely validated for mortality prediction across ischemic and non-ischemic cardiogenic shock cohorts [6,7]. However, this system does not account for underlying etiology, which may significantly influence prognosis and treatment response. Later, the Shock Academic Research Consortium (SHARC) proposed consensus definitions for cardiogenic shock (CS) classification based on etiology, categorizing patients into acute myocardial infarction-related CS (AMI-CS), heart failure-related CS (HF-CS), secondary-CS, and post-cardiotomy-CS [8]. While modern registry data demonstrated the feasibility of applying these definitions [9], comprehensive evaluation of all SHARC classes, particularly post-cardiotomy CS and secondary CS, remains limited. One study has evaluated the prognostic value of SCAI in cardiac surgical patients, but most studies evaluating CS classification have relied on databases, single-center cohorts, and focused primarily on AMI-CS and HF-CS [[10], [11], [12], [13]]. Furthermore, the comparative prognostic value of etiological versus severity-based classification systems has not been evaluated in contemporary populations receiving update CS management [9,11,12]. To date several scores has been developed to predict mortality in CS populations [14]. But these severity-based scores focus on the question: how sick is this patient? We hypothesized that etiological classification (SHARC) would provide incremental prognostic discrimination beyond severity staging (SCAI) across the full spectrum of CS etiologies. This represents a conceptual shift from severity-based assessment toward multidimensional risk stratification incorporating both etiology and severity. Thus, our aim is to evaluate a complementary question: does understanding the cause of the failing heart provide prognostic value beyond how sick the patient is? Given that, we made the hypothesis that adding etiological classification to severity classification, by capturing the underlying physiopathology may provide independent prognostic that align with current trends toward multidimensional phenotyping in critical illness.

This study aimed to evaluate the association between the SHARC etiological classification and in-hospital mortality. Additionally, we sought to assess the incremental prognostic value of combining SHARC classification with SCAI severity staging and hemodynamic parameters for risk stratification, and to characterize the epidemiological profiles, management patterns, and risk factors for mortality across SHARC classes.

## Material and methods

### Study design and population

The study was a retrospective, bicentric cohort including all consecutive adult patients (≥18 years) admitted to the cardiac Intensive Care Units (ICU) of Amiens-Picardie University Hospital and Dijon University Hospital between January 2018 and December 2023 for at least Stage B cardiogenic shock according to the SCAI classification [6]. Patients were included on the day of ICU admission. Patients were excluded if the diagnosis of cardiogenic shock was subsequently ruled out in favor of alternative etiologies such as hemorrhagic shock, refractory cardiac arrest. Other exclusion criteria were incomplete clinical or hemodynamic data precluding SHARC and SCAI classifications, terminal illness, legal guardianship or conservatorship, significant missing data (>10%), or loss to follow-up during hospitalization.

### Ethical considerations

The study was conducted in accordance with the principles of the Declaration of Helsinki and approved by the Institutional Review Board of Amiens University Hospital (PI2025_843_0187). Given its retrospective and non-interventional nature, the requirement for written informed consent was waived under French regulations. This report adheres to the STROBE (Strengthening the Reporting of Observational Studies in Epidemiology) statement for retrospective cohort studies [15].

### Definition, Staging and etiological classification of Cardiogenic Shock

Cardiogenic shock was defined according to international guidelines as clinical and biological evidence of tissue hypoperfusion secondary to cardiac dysfunction [8], meeting the criteria for at least SCAI Stage B. SCAI shock stages were determined at the time of ICU admission based on clinical presentation, laboratory findings, and hemodynamic parameters, ranging from Stage B (at risk) to Stage E (extremis with circulatory collapse). Stage B was defined as the presence of relative hypotension or tachycardia without overt hypoperfusion. Patients admitted with Stage C (classic CS), Stage D (deteriorating CS), or Stage E (extremis) were also included in the analysis [16,17]. SCAI stages were determined at the time of ICU admission and reassessed at 24 h. We calculated the CardShock score as described by Harjola et al. [18]

For this study, patients were retrospectively categorized into four etiological groups based on the primary cause of shock at ICU admission, according to the SHARC classification: acute myocardial infarction–CS (acute coronary syndrome with or without percutaneous coronary intervention), heart failure–related CS (de novo heart failure or acute decompensation of chronic heart failure), secondary-CS (resulting from valvular heart disease, arrhythmias, myocarditis, pulmonary embolism, pericardial disease, toxic, infectious, metabolic, endocrine, or inflammatory process) [19], and post-cardiotomy CS (after cardiac surgery requiring cardiopulmonary bypass) [8]. SHARC classification assignments were performed by two independent investigators (PGG and CB). Discrepancies were resolved by consensus with a third senior investigator (TH).

### Outcomes

The primary outcome was 30-day all-cause mortality, defined as death from any cause occurring within 30 days after ICU admission and classified according to the SHARC framework. Mortality data were obtained from hospital records and checked, when available, using national death registries.

The secondary outcomes were evaluated during the hospital stays, and are as follows: stroke, atrial/ventricular arrythmias, VA-ECMO, intra-aortic balloon pump use, acute kidney injury by KDIGO criteria, need for renal replacement therapy, infections, digestive ischemia and ICU-hospital length of stay.

### Data collection

Clinical, biological, treatments, ICU and hospital data were collected retrospectively in a computerized database. We performed computerized data extraction from our local data systems, DxCare® (DxCare®, Medasys), Diane Rea® (Bow Medical, France), Clinisoft® (Centricity Critical Care Clinisoft®, GE Healthcare), and ICCA® (Intelispace Critical care and Anesthesia®, Philips). Baseline characteristics included demographics, comorbidities, medications, and CS etiology. Right ventricular dysfunction was defined as TAPSE < 17 mm and/or fractional area change <35%. Clinical parameters at admission and 24 h included vital signs, laboratory values (lactate, pH, base excess), left ventricular ejection fraction, right heart failure, and hemodynamic measurements when available. Management variables included use of vasopressors/inotropes (type, dose), mechanical circulatory support (veno-arterial ECMO, intra-aortic balloon pump (IABP)), mechanical ventilation, and renal replacement therapy.

The Vasoactive-Inotropic Score (VIS) was calculated as: dobutamine dose (μg/kg/min) + dopamine dose (μg/kg/min) + 100 × epinephrine dose (μg/kg/min) + 100 × norepinephrine dose (μg/kg/min) + 10,000 × vasopressin dose (U/kg/min) + 10 × milrinone dose (μg/kg/min) [20].

Norepinephrine equivalent dose (NEE) was calculated according to Kotani et al. [21] by using the following formula: NEE = Norepinephrine dose (μg kg^−1^ min^−1^) + Epinephrine dose (μg kg^−1^ min^−1^) + 1/100 × Dopamine dose (μg kg^−1^ min^−1^) + 1/2.2 × Phenylephrine dose (μg kg^−1^ min^−1^) + 2.5 X Vasopressin (ui min^−1^). Norepinephrine is norepinephrine bitartrate expressed as μg kg^−1^ min^−1^ [21].

### Statistical analysis

Continuous variables were expressed as medians with interquartile ranges (IQR) or means with standard deviations, according to their distribution assessed by the Shapiro–Wilk test. Categorical variables were reported as numbers (n) and percentages (%). Between-group comparisons were performed using the Mann–Whitney U test for continuous variables and the chi-square or Fisher’s exact test for categorical variables, as appropriate. Variables with more than 10% missing values were not included in the analysis. Otherwise, missing data were imputed using multiple imputation by chained equations with predictive mean matching. The five imputed datasets were subsequently pooled according to Rubin's rules.

Associations between 30-day all-cause mortality and clinical, hemodynamic, and treatment-related variables were evaluated using two methods. The first method was based on the calculation of the risk ratio (RR), and the second method on multivariate logistic regression analyze. We focused on demographic variables before admission (age, genre, comorbidities, chronic medications), hemodynamic variables at admission to ICU (mean arterial pressure, heart rate, central venous pressure, LVEF, right ventricular dysfunction), hemodynamic support at admission (type of vasopressor/inotrope, VIS, number of catecholamines), and SCAI stage at admission to ICU. Variables with a p-value <0.20 in univariate analysis or strong clinical relevance were entered into the multivariable model. Backward stepwise elimination (retention threshold p < 0.10) was applied to identify independent predictors of mortality. The interaction between SCAI and SHARC classifications was included in the model. Model calibration was assessed using the Hosmer–Lemeshow goodness-of-fit test and model discrimination was evaluated using the C-statistic. Collinearity between variables was assessed by calculating VIFs. Results were expressed as odds ratios (ORs) with 95% confidence intervals (CIs).

To assess the prognostic performance of the SHARC and SCAI classifications for predicting 30-day mortality, we performed a stratified analysis to avoid collinearity between classifications and their components. Given that SCAI classification is derived from biological variables and vasopressor use, we constructed sequential nested logistic regression models to evaluate true incremental value. First, we tested SCAI's contribution by comparing models with and without its individual components (lactate, vasopressor use, hemodynamic parameters). Second, we evaluated SHARC's incremental value beyond all clinical predictors. The baseline model included established mortality predictors identified in our multivariable analysis (age, pH, chronic renal failure, right ventricular dysfunction, cardiac arrest, hemodynamic variables and *acute/chronic* cardiovascular medications). Model performance was compared using the Akaike Information Criterion (AIC) [22], with lower values indicating better model fit, and likelihood ratio χ² tests to assess statistical significance of incremental prognostic value. We specifically tested: (1) SCAI classification without hemodynamic variables versus baseline model, (2) SCAI classification added to a model including hemodynamic variables, and (3) SHARC classification added to the full clinical model. Complementary analyses included Bayesian Information Criterion (BIC) and Nagelkerke pseudo-R² to confirm robustness of findings. Sensitivity analyses were performed to assess robustness of the main model, and the interaction between SCAI and SHARC classifications.

To complement the information-criterion analysis, we compared discrimination using ROC curves for SHARC, admission SCAI, SHARC + SCAI, CardShock, admission VIS, M7 (baseline + hemodynamics + vasopressors + SHARC), and M9 (M7 + SCAI). AUCs with 95% CIs were estimated by DeLong’s method; optimal cut-offs by Youden’s J; and sensitivity, specificity, and predictive values with two-sided Wilson 95% CIs. Pairwise AUCs were compared using DeLong tests.

A sensitivity analysis based on a mixed-effects logistic regression including a random intercept for center tested for potential inter-site clustering was conducted to evaluate consistency of associations across type of cardiogenic shock and shock severity levels. A sensitivity analysis using a mixed-effects logistic regression model with a random intercept for center was conducted to account for potential inter-site clustering and to assess the consistency of associations across cardiogenic shock types and severity levels. All tests were two-tailed, and a p-value <0.05 was considered statistically significant. Analyses were conducted using R software (R Foundation for Statistical Computing, Vienna, Austria; version 4.3.0) with RStudio.

## Results

### Study population and baseline characteristics

**Fig. 1:**
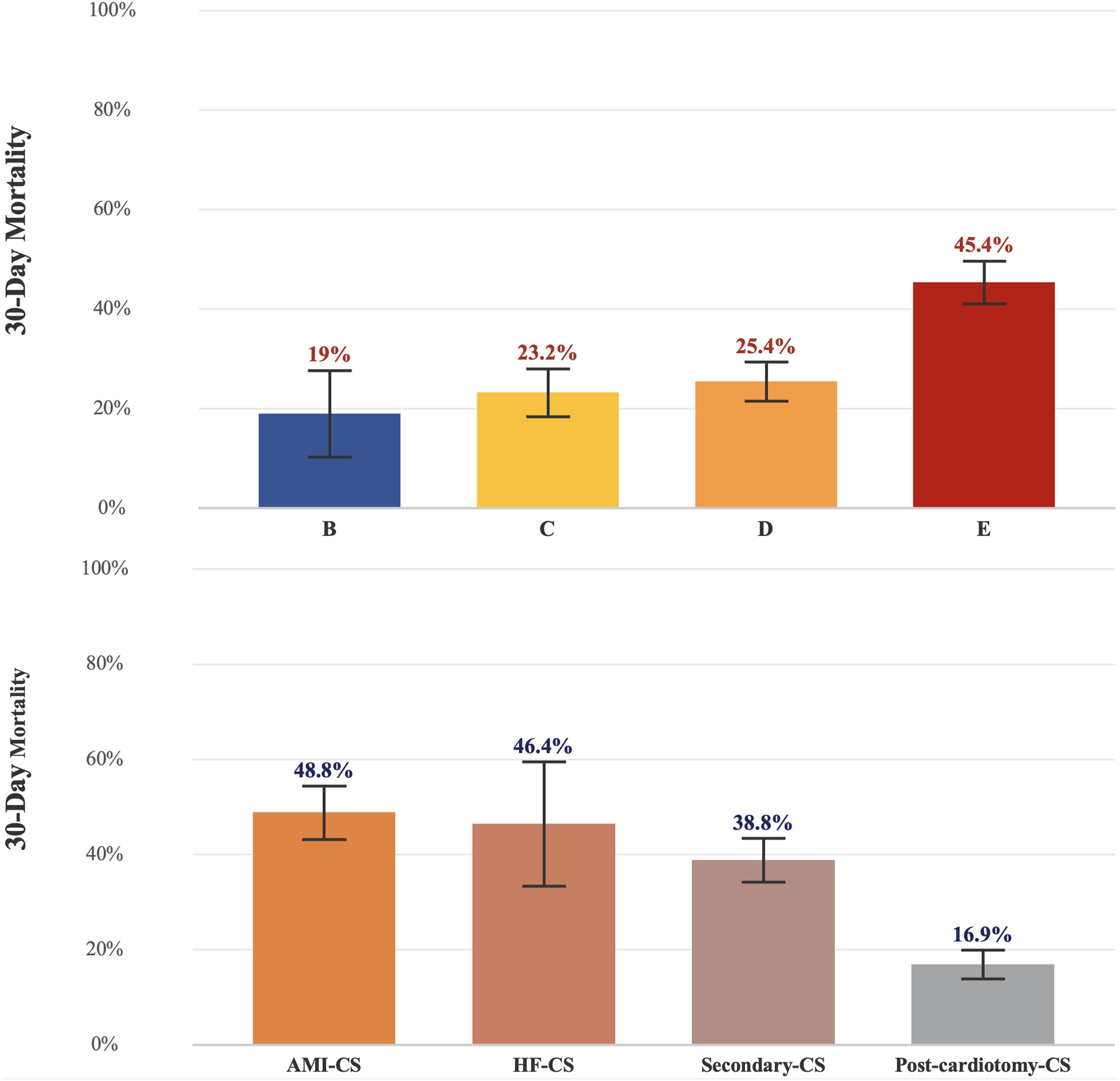
Distribution of mortality according to SHARC and SCAI classification in the overall cohort.

Among 1570 patients screened, 1366 met inclusion criteria and were analyzed (ESM-flow chart diagram). The distribution across SHARC classes was: AMI-CS (n = 303, 22.5%), HF-CS (n = 56, 5.0%), secondary-CS (n = 433, 29.9%), and post-cardiotomy-CS (n = 574, 42.7%) (Fig. 1). Baseline characteristics differed significantly across SHARC classes (Table 1, Supplementary Table S1).

**Table 1:** Baseline characteristics by SHARC Classification. Demographic, and medical data according to treatment group.

Variables	AMI-CS *N = 303*	HF-CS *N = 56*	Secondary-CS *N = 433*	Post-cardiotomy-CS *N = 574*	p-value
Age (years)	67.0 [58.0; 74.3]	65.4 [60.3; 72.2]	68.0 [56.8; 74.8]	70.0 [63.7; 76.0]	<0.001
Male, n (%)	206 (68.2%)	39 (70.9%)	273 (63.3%)	402 (70.0%)	0.238
Boby mass index (kg/m^2^)	26.0 [23.7; 29.4]	26.2 [23.1; 29.3]	26.1 [22.9; 30.4]	26.5 [23.6; 30.1]	0.210
Last known LVEF (%)	45.0 [35.0; 55.0]	41.0 [33.8; 50.8]	40.0 [25.0; 50.0]	55.0 [40.0; 60.0]	<0.001
Aspirin, n (%)	74 (24.4%)	20 (35.7%)	127 (29.3%)	260 (45.3%)	<0.001
P2Y12 platelet inhibitors, n (%)	17 (12.8%)	5 (8.93%)	14 (6.93%)	43 (7.49%)	0.062
Angiotensin-converting enzyme, n (%)	80 (26.4%)	11 (19.6%)	137 (31.6%)	193 (33.6%)	0.040
Valsartan/sacabutril, n (%)	4 (1.32%)	6 (10.7%)	24 (5.54%)	16 (2.79%)	0.002
Beta-blocker agent, n (%)	78 (25.7%)	27 (48.2%)	172 (39.7%)	349 (60.8%)	<0.001
Amiodarone, n (%)	3 (2.26%)	2 (3.57%)	10 (4.95%)	45 (7.84%)	0.063
Loop diuretic, n (%)	36 (11.9%)	24 (42.9%)	121 (27.9%)	221 (38.5%)	<0.001
Aldosterone anta-agonist, n (%)	13 (4.29%)	7 (12.5%)	64 (14.8%)	45 (7.84%)	<0.001
SGLT2 inhibitors, n (%)	1 (0.33%)	4 (7.14%)	8 (1.85%)	49 (8.58%)	<0.001
Statin, n (%)	69 (51.9%)	32 (57.1%)	86 (42.6%)	305 (53.1%)	0.015
Stroke, n (%)	24 (7.92%)	7 (12.5%)	39 (9.03%)	50 (8.71%)	0.730
Diabetes					
Non insulin	54 (17.8%)	11 (19.6%)	90 (20.8%)	63 (11.0%)	0.025
Insulin	7 (2.31%)	4 (7.14%)	19 (4.39%)	29 (5.05%)	
Both	17 (5.61%)	4 (7.14%)	16 (3.70%)	19 (3.31%)	
Dyslipidemia, n (%)	122 (40.4%)	32 (57.1%)	179 (41.3%)	281 (49.0%)	0.008
Chronic obstructive pulmonary disease, n (%)	24 (7.92%)	5 (8.93%)	41 (9.47%)	50 (8.71%)	0.912
Chronic high blood pressure, n (%)	163 (53.8%)	29 (51.8%)	225 (52.0%)	388 (67.6%)	<0.001
Atrial arrythmia, n (%)	28 (9.24%)	17 (30.4%)	126 (29.1%)	174 (30.3%)	<0.001
Chronic renal failure (eGFR <60 (mL min^−1^ m^−2^), n (%)	29 (9.57%)	8 (14.3%)	71 (16.4%)	143 (24.9%)	<0.001
Dialysis, n (%)	1 (0.33%)	1 (1.79%)	8 (1.85%)	18 (3.14%)	0.027
Previous cardiac surgery, n (%)	10 (3.32%)	4 (7.14%)	26 (6.00%)	189 (32.9%)	<0.001
Pre-admission cardiac arrest, n (%)	136 (44.9%)	4 (7.14%)	105 (24.2%)	7 (1.22%)	<0.001
SCAi classification at admission					
B	10 (3.30%)	3 (5.36%)	28 (6.47%)	38 (6.62%)	
C	31 (10.2%)	5 (8.93%)	105 (24.2%)	152 (26.5%)	<0.001
D	88 (29.0%)	20 (35.7%)	114 (26.3%)	254 (44.3%)	
E	174 (57.4%)	28 (50.0%)	186 (43.0%)	130 (22.6%)	
Cardshock	4 [3–5]	2 [2–4]	3 [1–3]	2 [1–3]	<0.001

p-value refers to comparison between groups.

LVEF: left ventricular ejection fraction, SCAI: Society for Cardiovascular Angiography and Interventions, SLGT2: sodium-glucose co-transporter 2.

### Primary outcome: 30-Day mortality

Thirty-day mortality varied significantly across SHARC classes: AMI-CS (48.7%), HF-CS (45.6%), secondary-CS (39.0%), and post cardiotomy-CS (17.3%) (*p* <  0.001) (Table 2, Supplementary Table S2). Risk ratio analyses across SHARC classifications demonstrated that mortality was significantly lower in post cardiotomy-CS compared with AMI-CS (RR = 0.36, 95% CI 0.26–0.51, *p* < 0.001), secondary-CS (RR = 0.44, 95% CI 0.35–0.54, *p* < 0.001), and HF-CS (RR = 0.35, 95% CI 0.28–0.43, *p* < 0.001). Conversely, mortality was markedly higher in AMI-CS and HF-CS compared with post cardiotomy-CS (RR = 2.89, 95% CI 2.33–3.58, *p* < 0.001 and RR = 2.75, 95% CI 1.97–3.84, *p* < 0.001, respectively). In addition, secondary-CS patients had slightly lower mortality than AMI-CS (RR = 0.79, 95% CI 0.67–0.94, *p* = 0.008), whereas differences between HF-CS and secondary-CS were not statistically significant (RR = 1.20, 95% CI 0.88–1.62, *p* = 0.31).

**Table 2:** Hemodynamic parameters and treatment, outcomes by SHARC Classification.

Variables	AMI-CS *N = 300*	HF-CS *N = 57*	Secondary-CS *N = 426*	Post-cardiotomy-CS *N = 583*	p-value
SAPS II	54.0 [39.0; 72.0]	57.0 [45.0; 74.5]	50.0 [36.0; 68.0]	42.0 [33.0; 53.0]	<0.001
SCAi classification at 24 h					
B	2 (0.66%)	2 (3.57%)	8 (1.85%)	7 (1.22%)	<0.001
C	24 (7.92%)	4 (7.14%)	96 (22.2%)	81 (14.1%)	
D	113 (37.3%)	28 (50.0%)	152 (35.1%)	357 (62.2%)	
E	164 (54.1%)	22 (39.3%)	177 (40.9%)	129 (22.5%)	
Concomitant sepsis, n (%)	51 (17.0%)	5 (8.77%)	104 (24.4%)	45 (7.72%)	<0.001
Mechanical ventilation, n (%)	179 (62.8%)	18 (34.0%)	185 (46.5%)	552 (95.2%)	<0.001
Hemodynamic parameters and treatments					
Number of vasopressor/inotrope	2.00 [1.00; 2.00]	2.00 [1.00; 3.00]	1.00 [1.00; 2.00]	2.00 [1.00; 2.00]	<0.001
Admission vaso-inotropic score	17.0 [0.00; 66.8]	5.00 [0.00; 29.0]	10.0 [0.00; 39.1]	10.1 [4.95; 18.3]	0.001
Maximal vaso-inotropic score	80.6 [19.6; 185]	67.9 [10.0; 167]	40.2 [5.00; 138]	30.0 [12.1; 91.1]	<0.001
Maximal Norepinephrine equivalent dose (μg kg^−1^ min^−1^)	0.84 [0.40; 1.80]	0.92 [0.40; 2.00]	0.75 [0.28; 1.88]	0.30 [0.13; 0.91]	<0.001
Dobutamine, n (%)	229 (75.6%)	35 (62.5%)	254 (58.7%)	431 (75.1%)	<0.001
Norepinephrine, n (%)	244 (80.5%)	39 (69.6%)	296 (68.4%)	508 (88.5%)	<0.001
Epinephrine, n (%)	62 (20.5%)	7 (12.5%)	52 (12.0%)	70 (12.2%)	<0.001
Vasopressin, n (%)	40 (13.0%)	17 (25.0%)	43 (10.5%)	18 (3.09%)	<0.001
Admission LVEF (%)	25.0 [15.0; 35.0]	25.0 [15.0; 37.5]	20.0 [15.0; 40.0]	40.0 [30.0; 50.0]	<0.001
Admission right ventricular dysfunction, n (%)	108 (35.6%)	36 (64.3%)	193 (44.6%)	239 (41.6%)	<0.001
Admission pulmonary hypertension, n (%)	13 (4.29%)	18 (32.1%)	62 (14.3%)	223 (38.9%)	<0.001
Admission pH	7.30 [7.19; 7.40]	7.38 [7.31; 7.45]	7.33 [7.24; 7.41]	7.32 [7.28; 7.37]	<0.001
Admission arterial lactates (mmol l^−1^)	4.45 [2.50; 8.40]	2.00 [1.30; 6.35]	3.30 [1.90; 6.80]	2.00 [1.50; 3.10]	<0.001
Complications					
Stroke, n (%)	15 (11.5%)	3 (5.26%)	13 (6.67%)	37 (6.35%)	0.223
Atrial arrythmia, n (%)	110 (36.7%)	22 (38.6%)	98 (23.0%)	224 (38.4%)	<0.001
Ventricular arrythmia, n (%)	61 (20.3%)	13 (22.8%)	42 (9.86%)	82 (14.1%)	<0.001
VA-ECMO, n (%)	136 (44.9%)	11 (19.6%)	36 (8.31%)	82 (14.3%)	<0.001
IABP, n (%)	6 (4.51%)	1 (1.79%)	1 (0.50%)	15 (2.61%)	0.016
Blood transfusion, n (%)	62 (46.6%)	13 (23.2%)	79 (39.1%)	276 (48.1%)	0.004
Acute kidney injury, n (%)	232 (76.6%)	46 (82.1%)	308 (71.1%)	273 (47.6%)	<0.001
Renal replacement therapy, n (%)	98 (32.7%)	21 (41.2%)	141 (33.4%)	87 (16.1%)	<0.001
30-day death, n (%)	148 (48.8%)	26 (46.4%)	168 (38.8%)	97 (16.9%)	<0.001

p-value refers to comparison between groups.

ECMO: extracorporeal membrane oxygenation, IABP: intra-aortic balloon pump, LVEF: left ventricular ejection fraction, ICU: intensive care unit, SAPSII: simplified acute physiologic score II, SCAI: Society for Cardiovascular Angiography and Interventions.

**Fig. 2:**
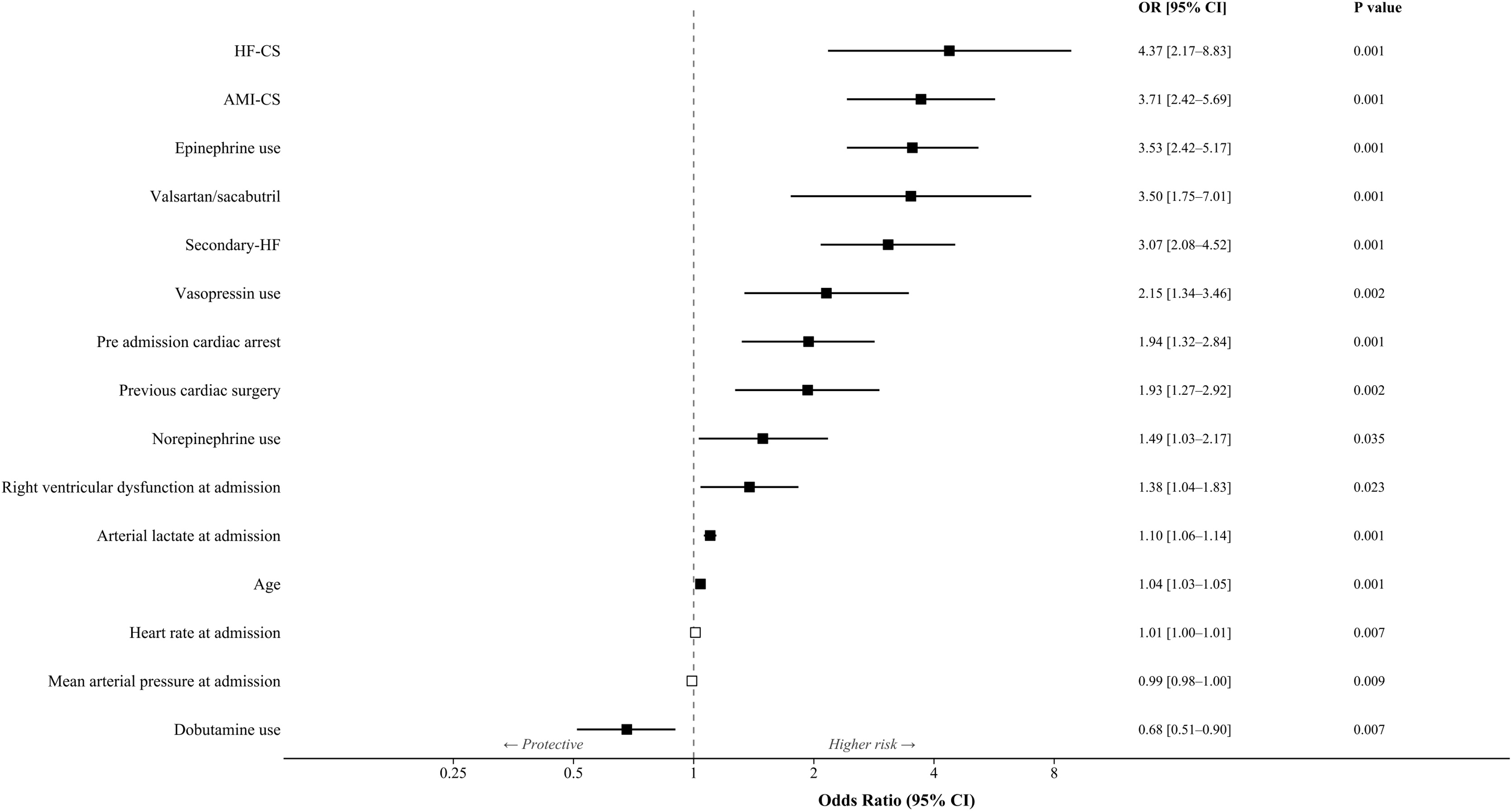
Forest plot of factors associated to 30-day mortality.

Multiple logistic regression demonstrated independent predictors of 30-day mortality comprise (Fig. 2, Supplementary Table S3) 14 variables such as comorbidities (age, prior cardiac surgery), chronic treatment (valsartan/sacubitril therapy), hemodynamic at ICU admission (arterial lactate, heart rate, mean arterial pressure, right ventricular dysfunction), hemodynamic treatment (norepinephrine use, epinephrine use, dobutamine use, vasopressin use), and the SHARC classification (AMI-CS (OR = 2.86, 95% CI 1.8–4.53, p < 0.001), HF-CS (OR = 3.95, 95% CI 1.96–7.96, p < 0.001), and secondary-CS (OR = 2.65, 95% CI 1.75–4.01, p < 0.001)). The OR are exposed in Supplementary Table S3 and range between 4.37 to 0.68. Collinearity was assessed by calculating VIF that ranged between 1.07 to 1.82. The final model demonstrated good discrimination with an AUC of 0.82 (95% CI 0.801−0.845) and adequate calibration (Hosmer-Lemeshow test, p = 0.195).

### Models’ performance

**Fig. 3:**
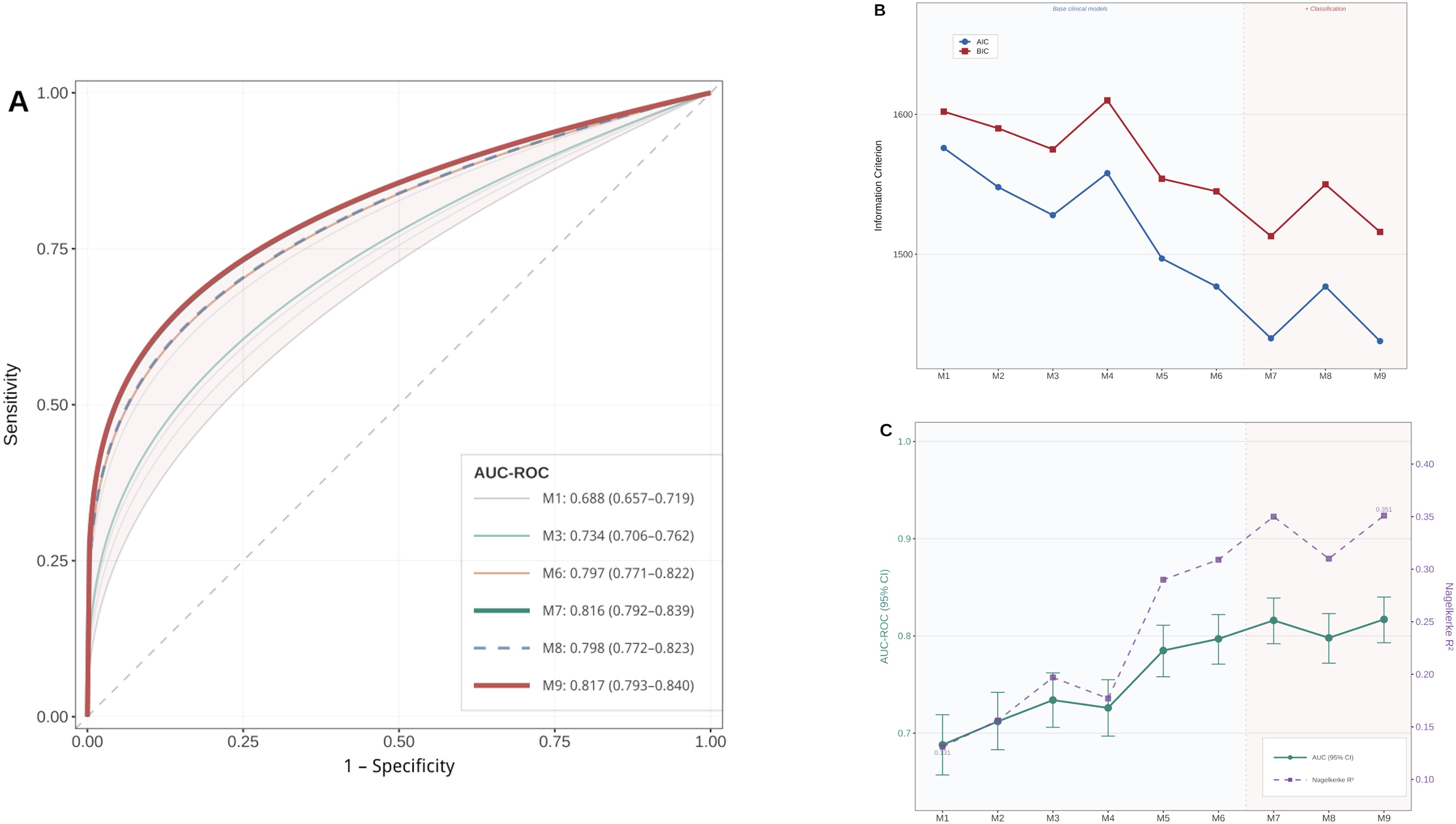
Incremental prognostic value of sequential logistic regression models (M1–M9) for in-hospital mortality. Each model builds progressively upon a baseline model (M1) by sequentially adding classification systems, hemodynamic parameters, and therapeutic variables to assess their incremental contribution to mortality prediction. Panel A: Receiver operating characteristic (ROC) curves comparing discriminative performance across all nine models. AUC, area under the ROC curve. Panel B: Changes in Akaike Information Criterion (AIC) and Bayesian Information Criterion (BIC) from M1 to M9. A decrease in AIC or BIC indicates improved model fit; the magnitude of decrease reflects the incremental prognostic contribution of each added variable set. Panel C: Progression of AUC and Nagelkerke R² from M1 to M9. An increase in AUC reflects improved discrimination, while an increase in R² reflects improved explained variance. Model composition: M1 = demographics + comorbidities (baseline model); M2 = M1 + SCAI staging; M3 = M1 + SHARC classification; M4 = M1 + hemodynamic parameters + arterial lactate + right ventricular dysfunction; M5 = M1 + inotrope and vasopressor therapy; M6 = M5 + hemodynamic parameters + arterial lactate; M7 = M6 + SHARC classification; M8 = M6 + SCAI staging; M9 = M8 + SHARC classification (full model).

Models’ performance is summarized in Supplementary Table S4 and Fig. 3. The baseline model (AUC = 0.703, R² = 0.148) showed modest discrimination. Adding the SCAI classification improved fit (ΔAIC = −20.0) and discrimination (AUC = 0.723, R² = 0.172). Inclusion of the SHARC classification provided a larger incremental gain (ΔAIC = −48.3; AUC = 0.734; R^2^ = 0.198), indicating greater prognostic information. Models incorporating hemodynamic and treatment variables achieved further improvements (AUC ≈ 0.80, R^2^ > 0.31). The combined model integrating SHARC, SCAI, and hemodynamic parameters showed the best performance (AUC = 0.819; R^2^ = 0.355), confirming their additive predictive value. Sensitivity analyses detailed in ESM confirmed the main analysis results, revealing no center effect and consistent mortality predictors across SHARC and SCAI subgroups.

At admission, SHARC achieved an AUC of 0.669 (95% CI 0.641–0.697), higher than SCAI (0.594, 0.563–0.624) and admission VIS (0.532, 0.499–0.565) (both p < 0.001). The SHARC + SCAI combination reached 0.687 (0.658–0.716). The CardShock score yielded 0.715 (0.675–0.755), comparable to SHARC + SCAI (p = 0.571). Pairwise DeLong tests showed that the full model M9 was higher than all admission-only scores (CardShock, VIS, SHARC, SCAI; all p < 0.001; Supplementary Table).

### Shock severity, hemodynamic management and outcomes

Left ventricular ejection fraction at admission differed significantly between SHARC classifications, showing the most severe dysfunction in AMI-CS and HF-CS, moderate dysfunction in secondary-CS, and higher values in post-cardiotomy-CS (25.0 [15.0–35.0] % and 25.0 [15.0–37.5] % vs 20.0 [15.0–40.0] % vs 40.0 [30.0–50.0] %, *p* < 0.001) (Supplementary Table S2). Post-cardiotomy-CS patients received less aggressive vaso-inotropic support, with lower maximal norepinephrine equivalent doses (0.24 [0.09–0.84] μg kg^−^¹^1^ min^−1^ vs 0.68 [0.11–1.76], 0.48 [0.00–1.51], and 0.35 [0.00–1.29]; *p* < 0.001). They had lower rates of VA-ECMO use (14.3 % vs 44.9 %, 19.6 %, and 8.3 %; *p* < 0.001). Acute kidney injury was less frequent in post-cardiotomy-CS (47.6 % vs 76.6 %, 82.1 %, and 71.1 %; *p* < 0.001), and renal replacement therapy was also used less often (16.1 % vs 32.7 %, 41.2 %, and 33.4 %; *p* < 0.001). Atrial arrhythmia occurred in 38.4 % of post-cardiotomy-CS patients compared with 36.7 %, 38.6 %, and 23.0 % in other groups (*p* < 0.001).

## Discussion

Our study validates SHARC etiological classes in a modern large cohort of CS patients admitted to tertiary ICUs. Our findings extend prior validation of SCAI by demonstrating that underlying etiology has incremental prognostic information beyond severity staging alone. The incremental prognostic performance of SHARC classification may reflect its ability to capture the specific pathophysiology driving shock, the type of hemodynamic support required, and the differential response to treatment.

### Clinical findings

Our findings align with and extend the SHARC consensus, which emphasized the need for comprehensive phenotyping beyond traditional classification approaches [7,9,23]. While cardiogenic shock registries have reported meaningful mortality stratification using SHARC definitions [23,24] or specific scores, our cohort demonstrated that etiological classification retained independent prognostic significance even after adjustment for SCAI staging, hemodynamic parameters, type of vasopressor, and clinical variables. Analysis of the progressive logistic model revealed that the variables contributing the greatest incremental discrimination were catecholamines, hemodynamic parameters, and SHARC classification, with SCAI staging contributing less substantially.

The present findings are consistent with previous studies assessing mortality risk factors in CS and suggest that shock etiology may function as a fundamental pathophysiological determinant of outcome, providing prognostic information complementary to hemodynamic severity [25]. Etiology itself and associated factors such as chronic medication exposure (sacubitril/valsartan, beta-blocker therapy) may influence the trajectory of shock and the response to acute treatment [9,26,27]. In this regard, the number and type of catecholamines and the clinical context of the cardiogenic shock all carry prognostic weight that may not be not captured by the SCAI classification alone or risk scores. The integration of comorbidity burden with acute catecholamine patterns offers a more refined pathophysiological assessment than categorical shock staging, potentially explaining why vasopressor type contributed more to model performance than SCAI staging. Nevertheless, the associations between epinephrine or vasopressin use and mortality may partly reflect confounding by indication, as these agents are preferentially administered in the most severe shock phenotypes; however, they also exert direct deleterious effects, as demonstrated in AMI-CS [28]. Vasopressor type may therefore reflect both a marker of severity and a modifiable therapeutic determinant. While SCAI classification remains valuable for rapid bedside staging, it does not capture more nuanced determinants such as baseline cardiac function, underlying pathophysiology (mixed versus isolated cardiogenic shock), chronic medication exposure, and their pharmacological interactions, each of which reflects distinct pathophysiological mechanisms and hemodynamic responses to acute circulatory treatment [18,19].

The concurrent validation of SHARC and SCAI, together with their interdependence, argues for their combined rather than isolated application [29]. Such complementarity is particularly valuable given the clinical and hemodynamic heterogeneity of cardiogenic shock (CS) [30]. Our findings provide quantitative support for this integrated approach: SCAI stage D in post-cardiotomy CS, for instance, carries a markedly different prognosis from stage D in acute myocardial infarction–related CS (AMI-CS), reinforcing the need for classification systems that combine severity with etiology [11,31]. This heterogeneity also helps reconcile the divergent results of trials evaluating temporary mechanical circulatory support (tMCS). The neutral effect of VA-ECMO in AMI-CS reported by ECLS-SHOCK, which predominantly enrolled SCAI-C patients, contrasts with the survival benefit of microaxial flow pumps observed in DANGER despite a comparable SCAI distribution [[31], [32], [33], [34]]. ECMO-CS, while restricted to more severe SCAI stages, included a more heterogeneous etiological population [35]. Across these trials, the optimal timing and modality of tMCS likely depend on both the underlying etiology and the SCAI stage. An integrated, physiology-based framework matching device-specific hemodynamic effects to individual patient pathophysiology should refine device selection and improve outcomes across the spectrum of CS presentations.

### The case example of post-Cardiotomy CS

Post-cardiotomy CS represented a good illustration of the importance of the underlying physiopathology over hemodynamic expression. These patients have the lowest mortality despite being older with higher prevalence of cardiovascular comorbidities. In this case, several factors may explain this paradox. Unlike other CS etiologies, post-cardiotomy-CS can often be anticipated based on preoperative risk factors (LVEF, pulmonary hypertension, chronic renal failure) and intraoperative events, allowing for preemptive optimization (type of cardioplegia, withdrawal of medications, surgery strategy). Patients are also more exposed to medication associated with better outcomes following cardiogenic shock such as beta-blocker therapy or SGLT-2 inhibitors [36,37]. The predominant mechanisms leading to cardiogenic shock (such as myocardial stunning, ischemia-reperfusion injury, and systemic inflammatory response) are more transient and may better respond to supportive care, contrasting with the irreversible myocardial damage from coronary occlusion or end-stage remodeling in chronic heart failure [38]. These patients receive immediate protocolized hemodynamic support in controlled settings with immediate access to mechanical support if needed, unlike the variable pre-hospital and emergency department delays in other cardiogenic shock forms [30]. Organ failures such as renal or hepatic dysfunction appear better controlled in post-cardiotomy-CS, likely reflecting early use of mechanical circulatory support in refractory cases. The distinct SCAI distribution, preserved LVEF (median 45% vs. 25-40% in other classes), and lower lactate levels indicate a more stable hemodynamic state despite significant compromise. These patients required less vaso-inotropic support yet achieved better outcomes, suggesting that aggressive escalation may not be routinely necessary when timely optimization is ensured. Accordingly, the magnitude of vasoactive-inotropic support does not reliably predict the benefit of temporary mechanical assistance [39].

### Clinical implications

Our results combined to literature support the continued evolution toward integrated, multiaxis classification systems based on models that combine etiological context (SHARC), severity grading (SCAI), phenotype (type of hemodynamic support, right side heart failure, pulmonary hypertension), and response to treatment (hemodynamic degradation, tissue hypoperfusion) [8,19,20,40]. At the bedside, a staged approach to CS can be implemented, beginning with an initial assessment using the SHARC etiological classification, which provides immediate context for therapeutic decision-making. Early risk stratification through SCAI staging helps anticipate the treatment pathway and guide medical orientation according to shock severity. The dynamic nature of SCAI staging enables serial reassessment and structured communication of a patient's evolving hemodynamic trajectory. However, patients presenting with identical SCAI stage D shock require fundamentally different management depending on SHARC etiology: urgent revascularization with early microaxial flow pump consideration and avoidance of epinephrine in AMI-CS, controlled hemodynamic support with watchful waiting for myocardial recovery in post-cardiotomy CS, and trigger-directed therapy (arrhythmia termination, thrombolysis, or diuretics) in secondary CS or HF-CS. In each scenario, SHARC etiology redirects the entire management pathway; from hemodynamic therapeutics choice and mechanical device to anticipated recovery trajectory and goals-of-care discussions. Beyond classification, a comprehensive phenotyping framework incorporating the severity of hemodynamic alterations (mean arterial pressure, right ventricular dysfunction, cardiac arrest), the type and intensity of pharmacological support (vasopressor and inotrope selection), and the degree of end-organ dysfunction (arterial lactate, renal and hepatic injury) may offer a pragmatic approach to individualized risk stratification [40,41]. Integrating these dimensions with etiological and severity-based classifications may help personalize CS management and inform future research aimed at defining optimal therapeutic strategies tailored to each etiology.

Our study has several strengths and limits.

The rates of events seem higher than those observed in some studies but our study has included all type of CS-patients admitted to tertiary ICU, not general CS patients managed across all care settings. In this sense the high rate of history of cardiac arrest, blood transfusion, or mechanical support is in line with studies specifically focusing on ICU. Recent studies evaluating mortality rate and risk factors in AMI-CS observed cardiac arrest rate between 29 and 40%, the same has been observed for mechanical support rate with ECMO-VA rate and blood transfusion [35,42]. The high cardiac arrest rate should be interpreted within the context of case severity: the predominance of SCAI D-E stages and high VA-ECMO utilization confirm that our cohort captured the most severe CS presentations, while less severe cases were likely managed in intermediate care units not included in our registry [6,17]. Similarly, the low prevalence of HF-CS likely reflects the same referral pattern, as most HF-CS patients present with lower acuity and may be managed outside tertiary cardiac ICUs. Despite these severity-driven complication rates, SHARC etiological classification independently discriminated mortality, reinforcing that etiology provides prognostic value beyond severity metrics and treatment intensity. External validation in broader CS populations is warranted to confirm the generalizability of these findings.

Several validated prognostic scores have been developed to assess mortality in CS, including the CardShock and IABP-SHOCK II risk scores [25]. However, these scores were primarily derived from AMI-CS populations and include variables (e.g., post-PCI TIMI flow grade) that are not applicable to all CS etiologies [41]. Their predictive performance is therefore reduced in more heterogeneous populations, particularly in non-AMI-CS settings [14]. Ortega-Hernández et al. showed that CardShock performance varies by etiology, whereas SCAI staging maintains prognostic value across causes [43]. Consistently, recent French expert recommendations endorse SCAI for standardized diagnosis and staging within a multidisciplinary framework [29].This variability in score performance supports our main finding that assessing CS severity alone is insufficient to fully capture prognosis.

The retrospective design introduces potential selection bias. We did not assess the several subcategories of each SHARC classification due to data limitations and number of cases which may have inflate the statistical risk [19]. However, the systematic application of SHARC classification across all four categories provides valuable insights into cardiogenic shock heterogeneity. Conversely, the strength of our study lies precisely in its heterogeneity. By including all CS etiologies admitted to ICUs, we provide a comprehensive picture of contemporary ICU CS management that complements more selected cohorts [23]. Moreover, the consistency of our results across sensitivity analyses supports our findings, and neither center effect nor model specification changed the main associations, indicating that SHARC etiological classification offers stable prognostic information. Post-hoc sample size analysis confirmed adequate statistical power, with 439 observed events yielding more than 20 events per predictor variable, while satisfying all criteria for minimal overfitting (required n = 439 vs. actual n = 1,366). Given our primary objective, the analytical approach was not designed to identify and describe all potential risk factors, whether established or novel, but rather followed methodological recommendations for comparing classification systems, which specify the assessment of incremental prognostic contribution using information criteria rather than variable selection for prediction optimization [22]. Lastly, the inclusion of post-cardiotomy patients who are systematically excluded from most registries, addresses a critical knowledge gap. The magnitude of SHARC's incremental contribution nonetheless reflects our tertiary case-mix (42.7% post-cardiotomy) and requires confirmation in independent cohorts. Our high rates of advanced mechanical support validate that we captured truly severe cases, strengthening the robustness of our mortality associations.

## Conclusions

In the present study, conducted in tertiary cardiac ICU populations with most severe cardiogenic shock, the SHARC classification provides complementary mortality discrimination compared to SCAI staging alone. Integration of both etiological and severity classifications may improve risk stratification, enable more personalized therapeutic approaches and inform the design of future phenotype-targeted interventions in cardiogenic shock.

## Contributions

PGG, CB, TH: Conceptualization, Formal Analysis, Writing; PGG, CB, OAA, TH, MN: Data curation, Data analysis; PGG, CB, TH, OAA, MN, LL, BB, YM Writing – review.

## Consent for publication

Not applicable.

## Ethics declarations

Ethics approval and consent to participate.

## Declaration of Generative AI and AI-assisted technologies in the writing process

Generative AI and AI-assisted technologies were used solely to correct grammar and spelling during the preparation of this manuscript. No content, data interpretation, or intellectual contribution was generated by AI.

## Funding

None.

## Data availability

The data underlying this article will be shared on reasonable request to the corresponding author.

## Declaration of competing interest

MN, PGG and OAA received fees for lecture from Baxter. PGG received fees for lecture from AOP, Medtronic, Edwards and Vygon. PGG is consultant for ABBOT. Other authors do not declare any conflict of interest.
