# Supplementary material for: Risk Stratification in Predicting Cardiogenic Shock 30-Day Mortality admitted to ICU: beyond the Prognostic Performance of SHARC and SCAI Classifications
Source: Ann Intensive Care. 2026 May 20;16:100090. doi: 10.1016/j.aicoj.2026.100090 (PMC13218115; doi:10.1016/j.aicoj.2026.100090)
Supplement: Supplementary file 1 [file mmc1.docx]

**Risk Stratification in Predicting Cardiogenic Shock 30-Day Mortality admitted to ICU: beyond the Prognostic Performance of SHARC and SCAI Classifications**

Guinot Pierre-Grégoire^1,2,3,4^, MD, PhD; Thomas Hanquiez^5^, MD, PhD, Maxime Nguyen^1,2,3,4^, MD, PhD; Osama Abou-Arab,^6^ Md, PhD, Laurent Leborgne^5^, MD; PhD; Yazine Majoub^6^ Md, PhD, Bélaïd Bouhemad^1,2,3,4^, MD, PhD, Christophe Beyls^6,7^, MD, PhD; For the PROMIX study group.

**Flow chart diagram of the study**

***
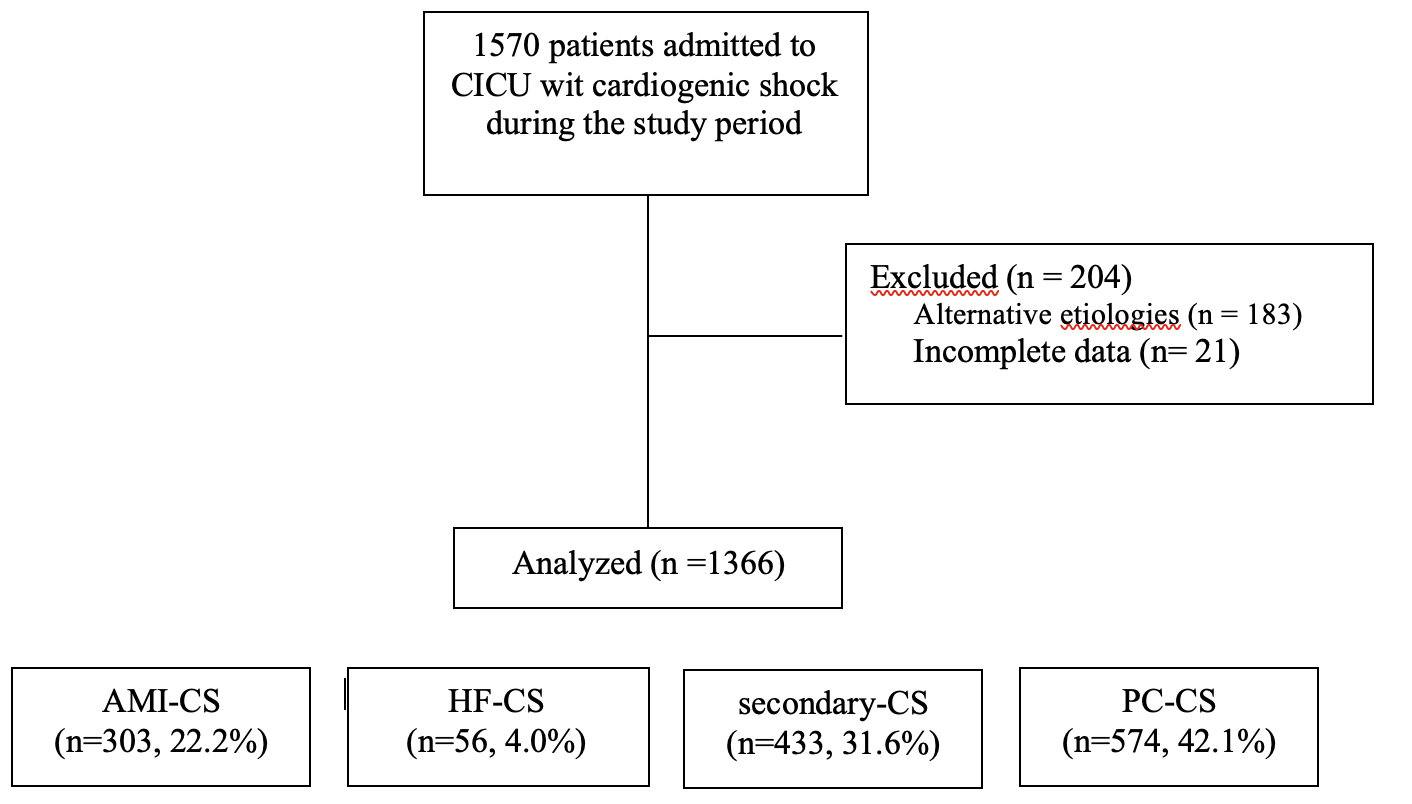
***

The causes of secondary -CS according were pulmonary embolism (n=69), Takotsubo syndrome (n=19), Non AMI post cardiac arrest (polytrauma, hypothermia, toxic)(n= 41), myocarditis (n=21), peripartum myocardiopathy (n=11), stress myocardiopathy (subarachnoid hemorrhage, perioperative, hemorrhagic shock…)(n=58), obstructive myocardiopathy (n=22), periprocedural cardiogenic shock (TAVI, atrial fibrillation ablation, post percutaneous pericardial synthesis…) (n=37), Chemotherapy induced (n=3), drug intoxication (n=23), sepsis related cardiac dysfunction (n=67), valvulopathy (n= 44), unclassified (n=18).

**Table of variables with missing data**

| **Variable** | **Number Missing** | **%** |
| --- | --- | --- |
| Last know LVEF | 210 | 15.3 |
| LVEF at admission | 83 | 6.1 |
| LVEF at 24 hours | 132 | 9.6 |
| BMI | 67 | 4.9 |
| Admission central venous pressure | 64 | 4.7 |
| Admission lactates | 50 | 3.7 |
| SAPS II | 34 | 2.5 |
| Admission heart rate | 35 | 2.6 |
| Admission systolic arterial pressure | 67 | 4.9 |
| Admission mean arterial pressure | 34 | 2.5 |
| Admisssion pH | 12 | 0.8 |
| Maximal NEE | 11 | 0.8 |
| Gender | 4 | 0.3 |
| Age | 1 | 0.1 |
| Dyslipidemia | 1 | 0.1 |

**Table 1.** Baseline characteristics by SHARC Classification. Demographic, and medical data according to treatment group. p-value refers to comparison between groups. LVEF: left ventricular ejection fraction, SCAI: Society for Cardiovascular Angiography and Interventions, SLGT_2_: sodium-glucose co-transporter 2.

| **Variables** | **AMI-CS**  ***N=303*** | **HF-CS**  ***N=56*** | **Secondary-CS**  ***N=433*** | **Post-cardiotomy-CS**  ***N=574*** | **p-value** |
| --- | --- | --- | --- | --- | --- |
| Age (years) | 67.0 [58.0;74.3] | 65.4 [60.3;72.2] | 68.0 [56.8;74.8] | 70.0 [63.7;76.0] | <0.001 |
| Male, n (%) | 206 (68.2%) | 39 (70.9%) | 273 (63.3%) | 402 (70.0%) | 0.238 |
| Boby mass index (kg/m^2^) | 26.0 [23.7;29.4] | 26.2 [23.1;29.3] | 26.1 [22.9;30.4] | 26.5 [23.6;30.1] | 0.210 |
| Last known LVEF (%) | 45.0 [35.0;55.0] | 41.0 [33.8;50.8] | 40.0 [25.0;50.0] | 55.0 [40.0;60.0] | <0.001 |
| Aspirin, n (%) | 74 (24.4%) | 20 (35.7%) | 127 (29.3%) | 260 (45.3%) | <0.001 |
| P2Y12 platelet inhibitors, n (%) | 17 (12.8%) | 5 (8.93%) | 14 (6.93%) | 43 (7.49%) | 0.062 |
| Angiotensin-converting enzyme, n (%) | 80 (26.4%) | 11 (19.6%) | 137 (31.6%) | 193 (33.6%) | 0.040 |
| Angiotensin II antagonist, n (%) | 22 (16.5%) | 7 (12.5%) | 25 (12.4%) | 112 (19.5%) | 0.066 |
| Valsartan/sacabutril, n (%) | 4 (1.32%) | 6 (10.7%) | 24 (5.54%) | 16 (2.79%) | 0.002 |
| Beta-blocker agent, n (%) | 78 (25.7%) | 27 (48.2%) | 172 (39.7%) | 349 (60.8%) | <0.001 |
| Amiodarone, n (%) | 3 (2.26%) | 2 (3.57%) | 10 (4.95%) | 45 (7.84%) | 0.063 |
| Loop diuretic, n (%) | 36 (11.9%) | 24 (42.9%) | 121 (27.9%) | 221 (38.5%) | <0.001 |
| Aldosterone anta-agonist, n (%) | 13 (4.29%) | 7 (12.5%) | 64 (14.8%) | 45 (7.84%) | <0.001 |
| SGLT2 inhibitors, n (%) | 1 (0.33%) | 4 (7.14%) | 8 (1.85%) | 49 (8.58%) | <0.001 |
| Statin, n (%) | 69 (51.9%) | 32 (57.1%) | 86 (42.6%) | 305 (53.1%) | 0.015 |
| Stroke, n (%) | 24 (7.92%) | 7 (12.5%) | 39 (9.03%) | 50 (8.71%) | 0.730 |
| Diabetes  Non insulin  insulin  both | 54 (17.8%)  7 (2.31%)  17 (5.61%) | 11 (19.6%)  4 (7.14%)  4 (7.14%) | 90 (20.8%)  19 (4.39%)  16 (3.70%) | 63 (11.0%)  29 (5.05%)  19 (3.31%) | .  0.025 |
| Dyslipidemia, n (%) | 122 (40.4%) | 32 (57.1%) | 179 (41.3%) | 281 (49.0%) | 0.008 |
| Obstructive Sleep Apnea Syndrome, n (%) | 12 (9.02%) | 4 (7.14%) | 21 (10.4%) | 58 (10.1%) | 0.879 |
| Chronic obstructive pulmonary disease, n (%) | 24 (7.92%) | 5 (8.93%) | 41 (9.47%) | 50 (8.71%) | 0.912 |
| Chronic high blood pressure, n (%) | 163 (53.8%) | 29 (51.8%) | 225 (52.0%) | 388 (67.6%) | <0.001 |
| Atrial arrythmia, n (%) | 28 (9.24%) | 17 (30.4%) | 126 (29.1%) | 174 (30.3%) | <0.001 |
| Ventricular arrythmia, n (%) | 13 (9.77%) | 16 (28.6%) | 51 (25.2%) | 30 (16.5%) | 0.001 |
| Coronary disease, n (%) | 46 (15.2%) | 15 (26.8%) | 50 (11.5%) | 220 (38.3%) | <0.001 |
| Valvular disease, n (%) | 13 (4.29%) | 23 (41.1%) | 59 (13.6%) | 342 (59.6%) | <0.001 |
| Peripheral arteriopathy, n (%) | 36 (11.9%) | 6 (10.7%) | 56 (12.9%) | 58 (10.1%) | 0.559 |
| Chronic renal failure (eGFR <60 (mL min^−1^ m^−2^), n (%) | 29 (9.57%) | 8 (14.3%) | 71 (16.4%) | 143 (24.9%) | <0.001 |
| Dialysis, n (%) | 1 (0.33%) | 1 (1.79%) | 8 (1.85%) | 18 (3.14%) | 0.027 |
| Smoke  active  old | 128 (42.2%)  43 (14.2%) | 23 (41.1%)  9 (16.1%) | 111 (25.6%)  83 (19.2%) | 157 (27.4%)  49 (8.54%) | <0.001 |
| Previous cardiac surgery, n (%) | 10 (3.32%) | 4 (7.14%) | 26 (6.00%) | 189 (32.9%) | <0.001 |
| Pre-admission cardiac arrest, n (%) | 136 (44.9%) | 4 (7.14%) | 105 (24.2%) | 7 (1.22%) | <0.001 |
| SCAi classification  B  C  D  E | 10 (3.30%)  31 (10.2%)  88 (29.0%)  174 (57.4%) | 3 (5.36%)  5 (8.93%)  20 (35.7%)  28 (50.0%) | 28 (6.47%)  105 (24.2%)  114 (26.3%)  186 (43.0%) | 38 (6.62%)  152 (26.5%)  254 (44.3%)  130 (22.6%) | <0.001 |

**Table 2.** Hemodynamic parameters and treatment, outcomes by SHARC Classification. p-value refers to comparison between groups. ECMO: extracorporeal membrane oxygenation, IABP: intra-aortic balloon pump, LVEF: left ventricular ejection fraction**,** ICU: intensive care unit, SAPSII: simplified acute physiologic score II, SCAI: Society for Cardiovascular Angiography and Interventions**.**

| **Variables** | **AMI-CS**  ***N=300*** | **HF-CS**  ***N=57*** | **Secondary-CS**  ***N=426*** | **Post-cardiotomy-CS**  ***N=583*** | **p-value** |  |
| --- | --- | --- | --- | --- | --- | --- |
| SAPS II | 54.0 [39.0;72.0] | 57.0 [45.0;74.5] | 50.0 [36.0;68.0] | 42.0 [33.0;53.0] | <0.001 |  |
| SCAi classification at 24 hours  B  C  D  E | 2 (0.66%)  24 (7.92%)  113 (37.3%)  164 (54.1%) | 2 (3.57%)  4 (7.14%)  28 (50.0%)  22 (39.3%) | 8 (1.85%)  96 (22.2%)  152 (35.1%)  177 (40.9%) | 7 (1.22%)  81 (14.1%)  357 (62.2%)  129 (22.5%) | <0.001 |  |
| Concomitant sepsis, n (%) | 51 (17.0%) | 5 (8.77%) | 104 (24.4%) | 45 (7.72%) | <0.001 |  |
| Mechanical ventilation, n (%) | 179 (62.8%) | 18 (34.0%) | 185 (46.5%) | 552 (95.2%) | <0.001 |  |
| *Hemodynamic parameters and treatments* | | | | | | |
| Number of vasopressor/inotrope | 2.00 [1.00;2.00] | 2.00 [1.00;3.00] | 1.00 [1.00;2.00] | 2.00 [1.00;2.00] | <0.001 |  |
| Admission vaso-inotropic score | 17.0 [0.00;66.8] | 5.00 [0.00;29.0] | 10.0 [0.00;39.1] | 10.1 [4.95;18.3] | 0.001 |  |
| Maximal vaso-inotropic score | 80.6 [19.6;185] | 67.9 [10.0;167] | 40.2 [5.00;138] | 30.0 [12.1;91.1] | <0.001 |  |
| Maximal Norepinephrine equivalent dose (µg kg^-1^ min^-1^) | 0.84 [0.40;1.80] | 0.92 [0.40;2.00] | 0.75 [0.28;1.88] | 0.30 [0.13;0.91] | <0.001 |  |
| Dobutamine, n (%) | 229 (75.6%) | 35 (62.5%) | 254 (58.7%) | 431 (75.1%) | <0.001 |  |
| Maximal dobutamine dose (µg kg^-1^ min^-1^) | 10.0 [5.00;10.0] | 10.0 [7.50;11.5] | 8.5[5.00;12.5] | 7.2 [5.00;10.0] | 0.005 |  |
| Norepinephrine, n (%) | 244 (80.5%) | 39 (69.6%) | 296 (68.4%) | 508 (88.5%) | <0.001 |  |
| Maximal norepinephrine dose (µg kg^-1^ min^-1^) | 0.68 [0.11;1.76] | 0.48 [0.00;1.51] | 0.35 [0.00;1.29] | 0.24 [0.09;0.84] | <0.001 |  |
| Epinephrine, n (%) | 62 (20.5%) | 7 (12.5%) | 52 (12.0%) | 70 (12.2%) | <0.001 |  |
| Maximal epinephrine dose (µg kg^-1^ min^-1^) | 0.39 [0.21;0.82] | 0.62 [0.33;1.30] | 0.46 [0.25;1.05] | 0.21 [0.11;0.89] | 0.015 |  |
| Vasopressin, n (%) | 40 (13.0%) | 17 (25.0%) | 43 (10.5%) | 18 (3.09%) | <0.001 |  |
| Maximal vasopressin dose (ui min^-1^) | 0.03 [0.02;0.03] | 0.03 [0.02;0.03] | 0.03 [0.01;0.04] | 0.03 [0.03;0.03] | 0.334 |  |
| Admission heart rate (BPM) | 94.0 [78.2;112] | 98.5 [74.8;118] | 100 [80.0;120] | 85.0 [77.2;99.0] | <0.001 |  |
| Admission systolic arterial pressure (mmHg) | 108 [92.0;124] | 102 [91.0;124] | 106 [92.0;121] | 108 [93.0;125] | 0.151 |  |
| Admission diastolic arterial pressure (mmHg) | 64.0 [53.0;75.0] | 60.0 [54.0;71.2] | 64.0 [53.0;75.0] | 59.0 [52.0;68.0] | <0.001 |  |
| Admission mean arterial pressure (mmHg) | 79.2 [67.4;90.3] | 75.0 [66.0;85.2] | 77.3 [66.7;89.7] | 75.2 [66.0;84.2] | 0.010 |  |
| Admission central venous pressure (mmHg) | 12.0 [9.00;15.2] | 17.5 [14.0;23.5] | 12.0 [5.00;15.0] | 11.0 [8.00;15.0] | 0.028 |  |
| Admission LVEF (%) | 25.0 [15.0;35.0] | 25.0 [15.0;37.5] | 20.0 [15.0;40.0] | 40.0 [30.0;50.0] | <0.001 |  |
| 24 hours LVEF (%) | 27.5 [16.2;35.0] | 20.0 [15.0;32.5] | 40.0 [30.0;50.0] | 45.0 [40.0;55.0] | <0.001 |  |
| Admission right ventricular dysfunction, n (%) | 108 (35.6%) | 36 (64.3%) | 193 (44.6%) | 239 (41.6%) | <0.001 |  |
| Admission pulmonary hypertension, n (%) | 13 (4.29%) | 18 (32.1%) | 62 (14.3%) | 223 (38.9%) | <0.001 |  |
| Admission pH | 7.30 [7.19;7.40] | 7.38 [7.31;7.45] | 7.33 [7.24;7.41] | 7.32 [7.28;7.37] | <0.001 |  |
| Admission arterial lactates (mmol l^-1^) | 4.45 [2.50;8.40] | 2.00 [1.30;6.35] | 3.30 [1.90;6.80] | 2.00 [1.50;3.10] | <0.001 |  |
| 24 hours arterial lactates (mmol l^-1^) | 2.40 [1.70;4.25] | 2.05 [1.70;2.98] | 2.00 [1.50;3.20] | 2.15 [1.60;3.48] | 0.008 |  |
| 24 hours fluid balance (ml) | 1200 [650;2100] | 575 [238;1100] | 1280 [620;2250] | 1200 [1000;2000] | <0.001 |  |
| *Complications* | | | | | | |
| Stroke, n (%) | 15 (11.5%) | 3 (5.26%) | 13 (6.67%) | 37 (6.35%) | 0.223 |  |
| Atrial arrythmia, n (%) | 110 (36.7%) | 22 (38.6%) | 98 (23.0%) | 224 (38.4%) | <0.001 |  |
| Ventricular arrythmia, n (%) | 61 (20.3%) | 13 (22.8%) | 42 (9.86%) | 82 (14.1%) | <0.001 |  |
| VA-ECMO, n (%) | 136 (44.9%) | 11 (19.6%) | 36 (8.31%) | 82 (14.3%) | <0.001 |  |
| IABP, n (%) | 6 (4.51%) | 1 (1.79%) | 1 (0.50%) | 15 (2.61%) | 0.016 |  |
| Blood transfusion, n (%) | 62 (46.6%) | 13 (23.2%) | 79 (39.1%) | 276 (48.1%) | 0.004 |  |
| Acute kidney injury, n (%) | 232 (76.6%) | 46 (82.1%) | 308 (71.1%) | 273 (47.6%) | <0.001 |  |
| KDIGO stage  1  2  3 | 51 (22.0%)  57 (24.6%)  124 (53.4%) | 10 (21.7%)  5 (10.9%)  31 (67.4%) | 71 (23.1%)  58 (18.8%)  179 (58.1%) | 120 (44.0%)  54 (19.8%)  99 (36.3%) | <0.001 |  |
| Renal replacement therapy, n (%) | 98 (32.7%) | 21 (41.2%) | 141 (33.4%) | 87 (16.1%) | <0.001 |  |
| Colitis or mesenteric ischemia, n (%) | 14 (10.5%) | 2 (3.57%) | 21 (10.4%) | 27 (4.70%) | 0.024 |  |
| 30-day death, n (%) | 148 (48.8%) | 26 (46.4%) | 168 (38.8%) | 97 (16.9%) | <0.001 |  |
| ICU length of stays (days) | 7.00 [3.00;16.0] | 5.00 [2.00;9.00] | 7.00 [3.00;15.0] | 4.00 [2.00;7.00] | <0.001 |  |
| Hospital length of stays (days) | 11.0 [5.00;22.2] | 12.0 [7.75;24.2] | 13.0 [6.00;24.0] | 11.0 [8.00;17.0] | 0.143 |  |

**Table 3.** Multivariable Analysis for 30-Day Mortality. Values are expressed as Odds ratio and their 95% confidence interval. Variables associated with 30 day-mortality in multivariate logistic regression. (CI95%)

| **Variable** | **Odds Ratio** | **IC 95%** | **Valeur p** |
| --- | --- | --- | --- |
| HF-CS | 4.37 | [2.17 - 8.83] | < 0.001 |
| AMI-CS | 3.71 | [2.42 - 5.69] | < 0.001 |
| Epinephrine use | 3.53 | [2.42 - 5.17] | < 0.001 |
| Valsartan/sacabutril | 3.50 | [1.75 - 7.01] | < 0.001 |
| Secondary-HF | 3.07 | [2.08 - 4.52] | < 0.001 |
| Vasopressin use | 2.15 | [1.34 - 3.46] | 0.002 |
| Pre admission cardiac arrest | 1.94 | [1.32 - 2.84] | < 0.001 |
| Previous cardiac surgery | 1.93 | [1.27 - 2.92] | 0.002 |
| Norepinephrine use | 1.49 | [1.03 - 2.17] | 0.035 |
| Right ventricular dysfunction at admission | 1.38 | [1.04 - 1.83] | 0.023 |
| Arterial lactate at admission | 1.10 | [1.06 - 1.14] | < 0.001 |
| Age | 1.04 | [1.03 - 1.05] | < 0.001 |
| Heart rate at admission | 1.01 | [1 - 1.01] | 0.007 |
| Mean arterial pressure at admission | 0.99 | [0.98 - 1] | 0.009 |
| Angiotensin-converting enzyme | 0.77 | [0.56 - 1.04] | 0.09 |
| Beta-blocker agent | 0.75 | [0.55 - 1.02] | 0.064 |
| Dobutamine use | 0.68 | [0.51 - 0.9] | 0.007 |
| pH at admission | 0.46 | [0.19 - 1.14] | 0.092 |

**Table 4. Model performance according to progressive logistic model anlaysis**

| **Model** | **Comparaison** | **AIC** | **Delta**  **AIC** | **BIC** | **Delta**  **BIC** | **R² Nagelkerke** | **AUC [IC95%]** | **Hosmer-Lemeshow test (p-value)** | **p-value** |
| --- | --- | --- | --- | --- | --- | --- | --- | --- | --- |
| M1: demographic + comorbidities |  | 1,558.9 | 0.0 | 1,590.2 | 0.0 | 0.148 | 0.703  (0.673-0.732) | 0.105 |  |
| M2: M1 + SCAI | M1 + SCAI | 1,538.9 | -20.0 | 1,585.7 | -4.5 | 0.172 | 0.723  (0.695-0.752) | 0.035 | 9.28e-06 |
| M3: M1 + SHARC | M1 + SHARC | 1,510.6 | -48.3 | 1,557.5 | -32.7 | 0.198 | 0.734  (0.707-0.762) | 0.595 | 9.44e-12 |
| M4: M1 + hemodynamic+ lactate+ right ventricular dysfunction | M1 + Hémo | 1,516.0 | -42.9 | 1,562.9 | -27.3 | 0.193 | 0.734  (0.706-0.763) | 0.081 | 1.34e-10 |
| M5: M1 + inotrope/vasopressor | M1 + hemodynamic+ lactate+ right heart failure | 1,380.4 | -178.5 | 1,442.9 | -147.3 | 0.313 | 0.799  (0.774-0.824) | 0.038 | <2e-16 |
| M6: M5 + hemodynamic + lactate | M5 + Hémo | 1,364.3 | -194.6 | 1,437.2 | -153.0 | 0.329 | 0.807  (0.782-0.831) | 0.013 | 4.21e-05 |
| M7: M6 + SHARC | M6 + SHARC | 1,338.0 | -220.9 | 1,426.5 | -163.7 | 0.354 | 0.818  (0.795-0.841) | 0.070 | 4.5e-07 |
| M8: M6 + SCAI | M6 + SCAI | 1,369.1 | -189.8 | 1,457.7 | -132.5 | 0.330 | 0.808  (0.783-0.832) | 0.002 | 0.769 |
| M9: M8 + SHARC | M8 + SHARC | 1,343.1 | -215.8 | 1,447.3 | -142.9 | 0.355 | 0.819  (0.796-0.842) | 0.008 | 5.14e-07 |

### 1. Mixed-Effects Logistic Regression

To account for potential inter-center variability, a mixed-effects logistic regression model was tested. The intraclass correlation coefficient (ICC) was 0.000 (0.0%), indicating no measurable clustering effect by center and confirming that outcomes were not center-dependent. Fixed effects remained stable, reproducing the main model’s findings.

| **Center** | **N patients** | **N décès** | **Mortality (%)** |
| --- | --- | --- | --- |
| 1 | 502 | 175 | 34.7 |
| 2 | 858 | 264 | 30.7 |

Independent predictors of 30-day mortality included:

| **Variables** | **Odds Ratio** | **IC 95%** | **p-value** |
| --- | --- | --- | --- |
| Age | 1.040 | [1.028 - 1.052] | 0.0000 |
| Valsartan/sacabutril | 3.584 | [1.771 - 7.254] | 0.0004 |
| Beta-blocker agent | 0.741 | [0.541 - 1.014] | 0.0611 |
| Prevous cardiac surgery | 1.895 | [1.225 - 2.931] | 0.0041 |
| Prior valvopathy (mitral, aortic, tricuspid) | 0.617 | [0.421 - 0.904] | 0.0131 |
| Previous cardiac surgery | 1.902 | [1.277 - 2.832] | 0.0016 |
| AMI-CS | 2.992 | [1.857 - 4.819] | 0.0000 |
| HF-CS | 4.109 | [1.998 - 8.451] | 0.0001 |
| Secondary-CS | 2.637 | [1.717 - 4.049] | 0.0000 |
| Norepinephrine | 2.861 | [1.211 - 6.761] | 0.0166 |
| Epinephrine | 6.702 | [2.790 - 16.098] | 0.0000 |
| Vasopressin | 4.229 | [1.714 - 10.435] | 0.0018 |
| Heart rate | 1.008 | [1.002 - 1.014] | 0.0070 |
| Mean arterial pressure | 0.990 | [0.982 - 0.998] | 0.0114 |
| Arterial lactate | 1.101 | [1.060 - 1.144] | 0.0000 |
| Right ventricular dysfunction | 1.400 | [1.044 - 1.876] | 0.0245 |

### 2. Subgroup Analyses by Etiology (SHARC Classification)

Subgroup analyses confirmed consistent directionality of associations across SHARC etiologies, although effect magnitudes varied with disease profile and sample size.

- **AMI-related CS:** Mortality was associated with age, chronic kidney disease, epinephrine exposure, and lactate elevation.
- **HF-related CS:** Age, epinephrine, vasopressin, ACE inhibitor therapy and pH were associated with mortality.
- **Secondary-CS:** Mortality correlated with age, cardiac arrest, epinephrine, vasopressin, and higher heart rate.
- **Post-cardiotomy CS:** Age, epinephrine, lactate, elevated CVP, right heart failure, beta-blocker therapy and higher MAP were associated with mortality.

### 3. Subgroup Analyses by SCAI Stage

Across all SCAI stages, mortality predictors were consistent, confirming the robustness of the main model. Catecholamine exposure, lactate, and age were the strongest drivers of mortality, while beta-blocker therapy remained protective.

- **Stage B (early shock):** Mortality associated with age, cardiac arrest, epinephrine, and lactate, beta-blockers.
- **Stage C (classic shock):** Entresto use, norepinephrine, epinephrine, and elevated lactate were independent risk factors.
- **Stage D (deteriorating shock):** Cardiac arrest, catecholamine exposure, and right heart failure and dobutamine.
- **Stage E (extremis):** Age, cardiac arrest, epinephrine, vasopressin, and lactate remained independently associated with death.

### 4. Overall Summary

Sensitivity analyses—including mixed-effects, etiologic, and stage-specific models—confirmed the stability of associations identified in the main analysis. There was no detectable center effect, and the key predictors (age, lactate, catecholamine exposure, vasopressin, and cardiac arrest) remained consistent across all subgroups. SHARC classification improved risk discrimination beyond hemodynamic and biochemical markers, whereas SCAI classification did not provide additional predictive value once these variables were incorporated. Protective effects of beta-blocker therapy and dobutamine persisted across all sensitivity analyses.

# ***Discriminative performance of admission scores and multivariable models for 30-day mortality***

# AUC and 95\% CI were computed using DeLong’s method. The optimal threshold was determined by Youden’s J statistic. Sensitivity, specificity, positive predictive value (PPV), and negative predictive value (NPV) were reported with two-sided Wilson 95% confidence intervals. Pairwise comparisons of AUCs were performed using DeLong’s test on the complete-case overlap of the two models being compared.

## Table. Panel Discriminative performance.

| **Models** | **AUC**  **(95% CI)** | **Best threshold** | **Se**  **(95% CI)** | **Sp**  **(95% CI)** | **PPV**  **(95% CI)** | **NPV**  **(95% CI)** |
| --- | --- | --- | --- | --- | --- | --- |
| **Admission stand-alone scores** |  |  |  |  |  |  |
| SHARC alone (univariate) | 0.669  (0.641-0.697) | 0.284 | 0.767  (0.727-0.803) | 0.516  (0.483-0.548) | 0.447  (0.413-0.482) | 0.813  (0.779-0.842) |
| SCAI alone (univariate) | 0.594  (0.563-0.624) | 0.377 | 0.468  (0.423-0.513) | 0.709  (0.679-0.737) | 0.451  (0.407-0.495) | 0.723  (0.693-0.751) |
| SHARC + SCAI (univariate combined) | 0.687  (0.658-0.716) | 0.331 | 0.707  (0.664-0.747) | 0.594  (0.562-0.626) | 0.471  (0.434-0.508) | 0.799  (0.767-0.827) |
| CardShock score | 0.684  (0.623-0.746) | 1.5 | 0.910  (0.826-0.956) | 0.348  (0.296-0.403) | 0.265  (0.216-0.321) | 0.938  (0.877-0.969) |
| Admission VIS | 0.532  (0.499-0.565) | 86.3 | 0.295  (0.255-0.338) | 0.841  (0.816-0.863) | 0.486  (0.428-0.544) | 0.700  (0.673-0.727) |
| **Multivariable models (manuscript Figure 3)** |  |  |  |  |  |  |
| M7 = baseline + hemodynamics + vasopressors + SHARC | 0.789  (0.757-0.821) | 0.228 | 0.802  (0.749-0.846) | 0.622  (0.583-0.659) | 0.467  (0.421-0.514) | 0.883  (0.850-0.910) |
| M9 = M7 + SCAI (full multivariable model) | 0.791  (0.759-0.822) | 0.248 | 0.770  (0.715-0.818) | 0.668  (0.630-0.704) | 0.490  (0.442-0.539) | 0.876  (0.843-0.902) |

##

## Table. Comparison between AUC by pairwise DeLong AUC comparisons

|  | **AUC A** | **AUC B** | **p-value**  **(DeLong)** |
| --- | --- | --- | --- |
| **Admission stand-alone, vs SHARC alone** |  |  |  |
| SHARC alone vs SCAI alone | 0.669 | 0.594 | < 0.001 |
| SHARC alone vs SHARC + SCAI | 0.669 | 0.687 | 0.014 |
| SHARC alone vs CardShock | 0.671 | 0.715 | 0.091 |
| SHARC alone vs admission VIS | 0.669 | 0.532 | < 0.001 |
| **Admission stand-alone, head-to-head** |  |  |  |
| CardShock vs admission VIS | 0.715 | 0.527 | < 0.001 |
| CardShock vs SHARC + SCAI | 0.715 | 0.699 | 0.571 |
| Admission VIS vs SHARC + SCAI | 0.532 | 0.687 | < 0.001 |
| **Best multivariable model (M9), vs other scores** |  |  |  |
| M9 vs CardShock | 0.810 | 0.721 | < 0.001 |
| M9 vs admission VIS | 0.791 | 0.537 | < 0.001 |
| M9 vs SHARC alone | 0.791 | 0.640 | < 0.001 |
| M9 vs SCAI alone | 0.791 | 0.612 | < 0.001 |
| M9 vs SHARC + SCAI | 0.791 | 0.672 | < 0.001 |
| M9 vs M7 (incremental contribution of SCAI) | 0.791 | 0.789 | 0.379 |
| **M7, vs admission stand-alone scores** |  |  |  |
| M7 vs CardShock | 0.808 | 0.721 | < 0.001 |
| M7 vs admission VIS | 0.789 | 0.537 | < 0.001 |
